# To Be or Not to Be?

**Published:** 1989-02

**Authors:** 


### Editorial

### To be or not to be?

Some explanaton and apology for the late arrival of this number is due to our readers. For the past 3 years this journal has enjoyed generous sponsorship from commercial sources. This ceased in 1988 and in 1989 the society had to face the doubling of its subscription, which had not been raised for many years, to keep the journal going. The sum involved was not large, nevertheless there were murmurings that there would be large scale resignations and it might be the end of the society. The committee felt that the only way to gauge opinion was to have a referendum of all members of the society. This was done, but it took time to organise and assess, hence this February number is 3 months late and appears at the same time as the May number.

Questionnaires were sent out to all members of the society asking them if they would consent to a doubling of their subscription in order that the journal might continue, 70% of those who replied agreed to accept a rise in subscription and half of these volunteered to make a donation to provide ready finance. Only 4 members took the opportunity of resigning, two of whom had moved away from Bristol and one was suffering from terminal illness. It must be said that only 40% of the questionnaires were returned, the editor found this disappointing, but those who know about these things said that this was a very good response, especially as no prepaid envelope had accompanied the questionnaire.

Here was a clear mandate to continue our journal. But there is more good news. The general airing of our problems has also brought us a new sponsor, The Nuffield Nursing Home Trust has now offered us generous financial support and we are back on the road again.

Perhaps the most important result of this period of self examination is the feeling that this journal which has, since its inception more than 100 years ago, aimed to be the 'medical journal of the south west' and has for the past three years been distributed to all doctors in the West of England, should now attempt to become a genuine *West of England Medical Journal*. If this is to succeed our society must ally itself with other West of England medical societies and specialist clubs and form a genuine partnership. An approach is being made to all these societies and together, with the approval and assistance of our new sponsors, we have hopes of launching in 1990, on a firm footing, a new *West of England Medical Journal*.

To return to our question and the musings of Hamlet. We have here '*an enterprise of great pith and moment*', we must not let its '*currents turn awry and lose the name of action*.'

